# Modification of Polyacrylonitrile Fibers by Coupling to Thiosemicarbazones

**DOI:** 10.3390/ma12233980

**Published:** 2019-11-30

**Authors:** Yao Yao, Yonghong Liang, Rahul Navik, Xiongwei Dong, Yingjie Cai, Ping Zhang

**Affiliations:** 1 National Local Joint Engineering Laboratory for Advanced Textile Processing and Clean Production, Wuhan Textile University, Wuhan 430200, China; yao19941126@163.com; 2 Hubei Provincial Engineering Laboratory for Clean Production and High-Value Utilization of Bio-based Textile Materials, Wuhan Textile University, Wuhan 430200, China; 1815073028@mail.wtu.edu.cn (Y.L.); rahul.navik2012@gmail.com (R.N.); yingjiecai@wtu.edu.cn (Y.C.)

**Keywords:** PAN fiber, surface modification, tyrosinase inhibitor, thiosemicarbazone, covalent coupling

## Abstract

This work reports the modification of Polyacrylonitrile (PAN) fibers by coupling to thiosemicarbazones to achieve the biological activity for the applications in the food product packaging. After modification, seven thiosemicarbazone compounds were synthesized. The as-synthesized thiosemicarbazone compounds were bonded to PAN fibers via covalent coupling, which was confirmed using Fourier transform infrared spectroscopy (FTIR), X-ray photoelectron spectroscopy (XPS) and scanning electron microscopy. The mean graft efficiency of the compounds was about 1.92%, and the antibacterial efficiency was 88.6% and 45.1% against Staphylococcus aureus (*S-aureus*) bacteria. All the seven thiosemicarbazone compounds exerted excellent tyrosinase activity, low cytotoxicity, excellent metal ion chelation ability, and anti-bacterial behavior against both gram-positive and negative bacteria. The mechanical properties of the fibers have been maintained without significant damage after the chemical modification. The break strength test and elongation at the break test were done to measure the fracture strength of the modified fibers. Overall, the promising properties of the modified PAN fibers show potential applications in food packaging materials for fruits and vegetables, which require long-term anti-browning effects during their transportation and storage.

## 1. Introduction

Thiosemicarbazone compounds have attracted great interests in the field of biology because they demonstrate a potential contribution in a wide array of pharmacological applications, such as anti-bacterial, fungal-proofing, and tyrosinase inhibitory effects. Also, they can be used for developing anti-melanogenic compounds in skin-whitening cosmetics products and browning resistant agents for foods [1]. Thiosemicarbazones compounds have pharmacological and biological properties to a large extent, because of the metal complexes, free ligands, and the position of substitutions which controls the reactivity. Thiosemicarbazone derivatives or macrocyclic compounds with different structures were formed by different substituents on the thiosemicarbazone and other characteristic organic substances [2].

In this study, seven different thiosemicarbazone compounds were synthesized using thiosemicarbazone and different substituents of benzaldehyde. The compounds not only showed low cytotoxicity and good metal ion chelation but also showed excellent biological activity and high efficiency. They inhibited tyrosinase activity of the modified PAN fibers finds potential applications [3].

Polyacrylonitrile (PAN) fibers are one of the most common synthetic fibers and have attracted considerable interests because of their unique properties, including low density, good thermal insulation, soft-feel, excellent mechanical properties, and high durability. PAN fibers have been widely used as a precursor for carbon fibers [4,5,6], or absorption substrates [7]. Many methods have been reported to modify the surface of PAN fibers, such as plasma treatment [8], ultrasonic impregnation [9], and biological enzymes treatment [10]. However, these techniques require pre-treatments, and the modification only last for short duration. Also, the enzyme treatment requires high purity of the enzymes which is challenging.

Tyrosinase is a copper-containing oxidoreductase which is widely distributed in microorganisms, animals, plants, and the human body. It plays a key role in melanin synthesis, which is the endogenous polyphenolic substance in oxidized fruits and vegetables [11,12]. It can easily cause a browning reaction in many fruits and vegetables during storage and processing, which results in severely damages to the nutrition, flavor, and appearance, leading to reduced quality and commodity value of fruit and vegetable products.

Based on the above research and innovative design, amino groups in the thiourea molecules were directly dehydrated on hydrolyzed PAN fibers via covalent grafting. The obtained composite fibers can inhibit tyrosinase activity and have good mechanical properties. The modified PAN fibers can be used as a storage and packaging material for fruits and vegetables to prevent its browning and to preserve fresh tastes [13,14].

## 2. Materials and Methods

### 2.1. Materials

PAN fiber, a synthetic fiber made from acrylonitrile copolymers of PAN or acrylonitrile content greater than 85% (mass percent), was kindly provided by Hangzhou wan PAN fiber Company Ltd., Hangzhou, China. All auxiliaries purchased were AR (analytical grade 99.9+ %) grade and supplied from commercial sources (e.g., Sigma, Shanghai, China). The auxiliaries were used directly without any further purification. To prepare the solutions and buffers, deionized water purified by a Millipore-Q ultra-purification system was used.

### 2.2. Synthesis Methods of Thiosemicarbazone Compounds

**Scheme 1:**
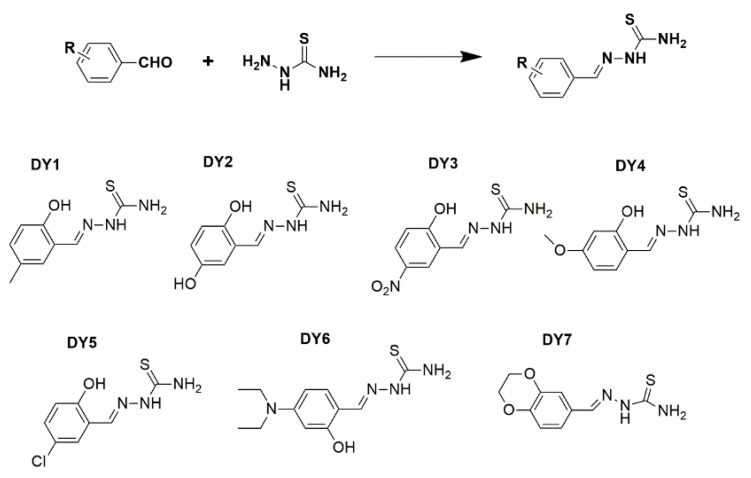
Synthesis route of thiosemicarbazones.

The thiosemicarbazone compounds were synthesized following a previously reported method [15], and the schematic equation is presented in Scheme 1. Atomistic investigations were conducted on a Perkin-Elmer 2400 analyzer (PerkinElmer, Waltham, MA, USA). The mass spectroscopy (MS) data were recorded with the help of a Quattro II ESI mass spectrometer (Waters, Massachusetts, USA), and the ESI-MS spectral data were presented in Table 1. In a typical synthesis method, firstly, 0.92 g of amino thiourea (approximately 10 mmol) was dissolved in 10 mL water. Then, an equimolar concentration of salicylaldehyde or its derivative was added in 20–30 mL of ethanol. After compounding, distilled water and a catalytic amount of glacial acetic acid were added to the solution and mixed well. The mixture was heated and refluxed for 2–5 h. Then, the mixture was cooled to room temperature. After standing overnight at 4 °C, the crude product was obtained by suction filtration, and the product was further recrystallized from 50% ethanol to give the products **DY1**–**DY7**.

### 2.3. ^1^HNMR (600 MHz) Spectra of the as Synthesized Thiosemicarbazone Compounds in Dimethyl Sulfoxide (DMSO) Presenting the Signals as Follows: (Chemical Shift in ppm, Varian Mercury 600 spectrometer)

**DY1**: δ 11.28 ppm (s, 1H), 9.55 (s, 1H), 8.33 (s, 1H), 8.03 (s, 1H), 7.85 (s, 1H), 7.70 (s, 1H), 7.01 (d, J = 6.8 Hz, 1H), 6.75 (d, J = 8.2 Hz, 1H), 2.21 (s, 4H).

**DY2**: δ 11.28 ppm (s, 1H), 9.12 (s, 1H), 8.73 (s, 1H), 8.30 (s, 1H), 8.00 (s, 1H), 7.71 (s, 1H), 7.19 (s, 1H), 6.68 (s, 2H).

**DY3**: δ 11.47 (s, 2H), 8.82 (s, 1H), 8.37 (s, 1H), 8.21 (s, 1H), 8.16 (s, 1H), 8.10 (d, J = 8.0 Hz, 1H), 7.05 (d, J = 9.1 Hz, 1H).

**DY4**: δ 11.18 (s, 1H), 9.90 (s, 1H), 8.27 (s, 1H), 7.92 (s, 1H), 7.76 (s, 2H), 6.46–6.39 (m, 2H), 3.73 (s, 3H).

**DY5**: δ 11.35 (s, 1H), 10.37 (s, 1H), 8.31 (s, 1H), 8.05 (s, 1H), 7.97 (d, J = 7.3 Hz, 1H), 7.91 (s, 1H), 6.92–6.83 (m, 2H).

**DY6**: δ 11.02 (s, 1H), 9.47 (s, 1H), 8.18 (s, 1H), 7.79 (s, 1H), 7.61 (s, 1H), 7.49 (s, 1H), 6.21 (d, J = 8.9 Hz, 1H), 6.09 (s, 1H), 1.10 (t, J = 6.8 Hz, 6H).

**DY7**: δ 11.23 (s, 1H), 8.01 (s, 1H), 7.91 (d, J = 16.3 Hz, 2H), 7.39 (s, 1H), 7.19 (d, J = 8.3 Hz, 1H), 6.86 (d, J = 8.3 Hz, 1H), 4.26 (d, J = 4.1 Hz, 4H).

### 2.4. Graft Modification

**Figure 1:**
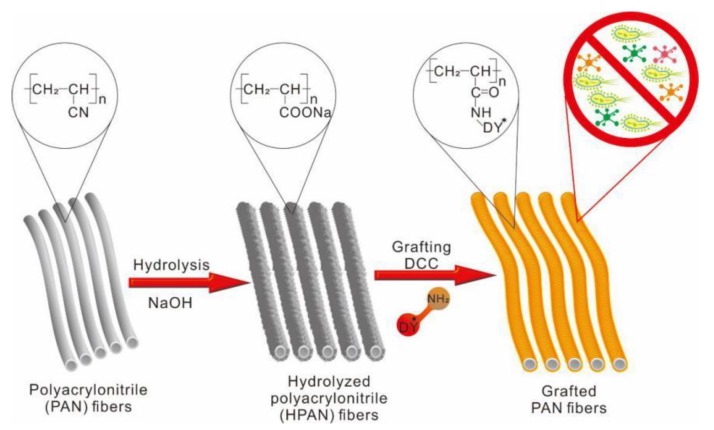
Grafting method and change in the chemical structure of PAN fibers.

A graphical representation of the grafting method and change in the chemical structure of the fibers after each stage is shown in Figure 1. The grafting was carried out in two steps: (1) hydrolysis of PAN fibers; and (2) grafting of thiosemicarbazone onto the hydrolyzed fibers produced as described above. First, 5.0 g of PAN fibers was added to 30 mL of 10% (*w*/*w*) NaOH aqueous solution, and the solution was stirred at 80 °C for 30–60 min to obtain the hydrolyzed polyacrylonitrile (HPAN) fibers. Then, the HPAN fibers were rinsed with deionized water until the water became neutral and then dried in an oven at 60 °C until a constant weight was obtained. The HPAN fibers were added to the as-prepared 2% thiosemicarbazone solution (using ethylene glycol as the solvent), and a half equimolar amounts of N, N’-dicyclohexylcarbodiimide (DCC) condensing agent was directly added. The grafting reaction was carried out at 80 °C for 2 h. Then, the grafted PAN fibers were separated, washed thoroughly with deionized water, and dried under vacuum at 50 °C.

### 2.5. Grafting Efficiency Calculation

Grafting efficiency (***G_e_***) can be calculated by Equation (1).

(1) $$G_{e}\%=\frac{m_{2}-m_{1}}{m_{1}}\times 100\%$$

where ***m*_1_** and ***m*_2_** are the mass of PAN fibers before and after thiosemicarbazone grafting, respectively. Measurements of each sample were conducted three times and the average value was used for result analysis.

### 2.6. Scanning Electron Microscope (SEM) Analysis

A scanning electron microscope (SEM, JSM-IT300, JEOL Ltd., Akishima, Japan) at 0.3–30 kV was used to examine the surface morphologies of PAN fibers before and after grafting modification.

### 2.7. FTIR Analysisg5g5

Infrared spectra of fibers samples with KBr pellets were obtained using FT-IR spectrometer (VERTEX 70 spectrometer, Bruker, Billerica, MA, USA).

### 2.8. Tyrosinase Activity Analysis

Tyrosinase activity was determined by spectrophotometry, as 3,4-dihydroxyphenylalanine (*L*-DOPA, Sigma) oxidation activity, with a modification of a reported method [16,17]. In brief, the tyrosinase solution (50 µL) was gently added to 450 µL of phosphate buffer at a concentration of 0.05 M containing 50 µM samples. The pH of the solution was maintained at 6.8. Then, the mixture was incubated at 30 °C for 10 min and 500 µL of 5 mM *L*-DOPA solution was added. It was noticed that the absorbance of the solution increased at 475 nm (ε = 3600 M cm^−1^) possibly due to the formation of *L*-DOPA chrome. This intensity of the peaks was recorded based on time. The tyrosinase activity was analyzed from the initial rate. The buffer containing 1% DMSO was used as a control sample. The inhibition rate of the test solution against tyrosinase was calculated using the corresponding negative control as a reference, and an approximation of the half-inhibitory concentration (IC_50_) was estimated based on the concentration-enzyme inhibition rate curve.

### 2.9. Electron Paramagnetic Resonance (EPR) Measurements

The EPR of Cu(II) and thiosemicarbazone compounds were recorded in liquid helium at 200 K temperatures on a Bruker A200 X-Band spectrometer (Bruker, Billerica, Mass., USA) with a 9.42-GHz field modulation equipped with a Bruker Instruments 4111VT helium flow cryostat (Bruker, Billerica, Mass., USA). **DY**–Cu (II) complexes were prepared by refluxing of Cu(NO_3_)_2_ and thiosemicarbazone compounds in ethanol taking 1:1 molar ratio for 4 h.

### 2.10. Tensile Fracture Strength Analysis

The mechanical properties of the fiber samples were tested on an electronic strength tester for a single fiber (LLY-06E/PC, Laizhou Electron Instrument Co., Ltd., Laizhou, China) and YG-003 fiber tensile machine (Changzhou Zhongxian Instrument Co., Ltd., Changzhou, China). The single fiber was clamped on a single fiber electron strength meter with a clamping length of 10–20 mm to stretch the sample. The stretching speed maintained to be 20 mm min^−1^ until the fibers were broken. The average data of 100 fibers samples were taken as the test result.

### 2.11. Determination of the Stability Constant of the Obtained Complexes

The stability constant of the complex was tested using a Metrohm 877 Titrino plus automatic potentiometric titrator (Metrohm, Herisau, Switzerland). The titration temperature was maintained at 25 ± 0.5 °C with pH range of 2–11. The titration solution was water solution with 20% DMSO, and the ionic strength *I* = 0.01 mol·L^−1^ KNO_3_ (aq). The initial concentration was [L] = 0.005–0.0001 mol·L^−1^, [HNO_3_] = 0.002 mol·L^−1^, [KNO_3_] = 0.10 mol·L^−1^, and [M] = 0.0001 mol·L^−1^. The total volume was 25 mL, the titration volume was 0.004 mL·D^−1^, and the response time was at least 1 min. The titration was triplicate and the titration data were fitted using Hyperquad 2013 software, where the ion product of H_2_O was pKw = 13.85 [18]. The distribution curve of species with pH was plotted using HySS 2009 software (Version 2009, Hyperquad Co. Ltd., London, UK) [19,20,21]. Three metal ions, Cu^2+^, Zn^2+^, and Mn^2+^, were chosen as model ions in this work. The stability constants of the thiosemicarbazone compounds forming complexes with the above three metal ions and the species distribution under different pH conditions were investigated in detail.

### 2.12. X-ray Photoelectron Spectroscopy (XPS)

X-ray photoelectron spectra were collected using a K-Alpha XPS system (ESCALAB 250Xi, Thermo Fisher Scientific, Waltham, USA) with a monochromatic Al Kα X-ray source. The XPS measurement was carried out to determine surface elemental stoichiometry by the sensitivity-factor-corrected peak-area ratios. The residual pressure inside the analysis chamber was in the 10^−9^ Pa range, the charge neutralizer filament was used during all experiments, and samples were sputter cleaned under vacuum (15 kV, 12 mA) to remove surface contamination. The peaks were collected with an analyzer pass energy of 30 eV at an interval of 1.00 eV.

### 2.13. The Cytotoxic Effect by 3-(4,5-Dimethylthiazol-2-yl)-2,5-diphenyltetrazolium bromide (MTT) Assays

The cytotoxic effect of compounds was determined by using in vitro colorimetric MTT assay [22]. The A549 cells were seeded into 96-well microtiter tissue culture plates with a final volume of 100 μL. After attachment for 24 h, the cells were treated with different concentrations (0.1, 1, 10, 50, and 100 μM) of thiosemicarbazone compounds in the culture medium. Eight replicate wells per concentration were used and all experiments were repeated in triplicate. The solvent control (DMSO) was also assayed in parallel. The cells were incubated for 24 h and 48 h with compounds. Then, MTT dye was added 4 h before the completion of the incubation periods. The resulting formazan crystals were separated from the medium and then dissolved in 100 μL DMSO. The absorbance at 490 nm was used to quantify the plates using ELx808 Absorbance Microplate Reader (Bio-Tek, Winooski, USA).

### 2.14. Antibacterial Test

Antibacterial performance against bacteria (*E. coli, S. aureus,* and *B. subtilis*) was determined according to GB/T20944.3-2008. The inoculation suspension of bacteria was prepared and the inoculation loop was used to conduct the bacterial inoculation on the agar board for 18–24 h at 37 ± 1 °C. The sample was dipped into the conical flask filled with experimental bacteria solution with a certain concentration. After that, the oscillation culture is conducted to the samples for specified time, and 1 mL of solution was taken, diluted and distributed onto an agar plate. All plates were incubated at 37 °C for 24 h and the colonies formed were counted. The antibacterial performance of the fiber was expressed in the inhibition rate (***R_i_***, Equation (2))

(2) $$R_{i}\%=\frac{W_{t}-Q_{t}}{W_{t}}\times 100\%$$

where ***W_t_*** is the bacteria-colony number of viable bacteria in the plate before antibacterial treatment or original PAN fibers. ***Q_t_*** is the bacteria-colony number of viable bacteria in the plate after antibacterial treatment or grafted PAN fibers.

## 3. Results

### 3.1. Determination of Tyrosinase Activity

**Figure 2:**
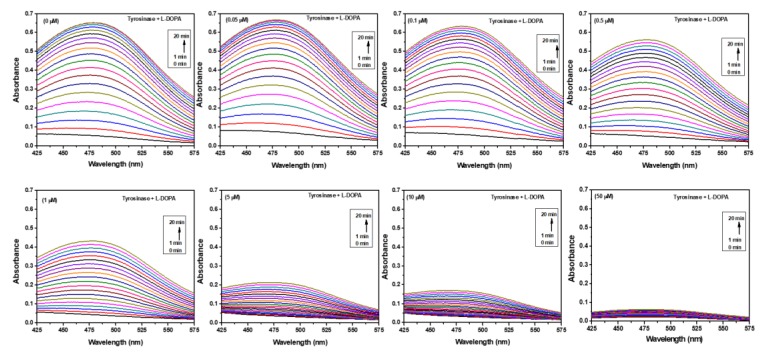
The effect of **DY6** inhibited tyrosinase activity as measured by the L-DOPA method.

**Figure 3:**
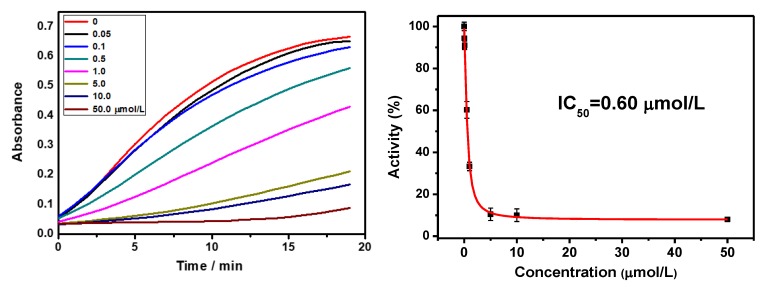
UV absorbance value versus time at 475 nm and semi-inhibitory IC_50_ curve of **DY6** (blank control reagent is 1% DMSO).

The UV absorption spectra of tyrosinase and thiosemicarbazone compound **DY1**–**DY7** were tested using the *L*-DOPA method. All compounds exhibited satisfactory activity, and the experimental results are shown in Figure 2. For comparison purposes, the absorbance at 475 nm as a function of scan time was plotted, as shown in Figure 3.

The results show that **DY6** significantly inhibited tyrosinase activity. The inhibition rate was positively correlated with the concentration, and the 50% inhibitory concentration (IC_50_) value of the semi-inhibitory concentration was 0.60 μM (**DY6**). The analysis shows that the compound has the effect of inhibiting the formation of melanin on the skin. To compare the cytotoxicity data, the concentrations of different chelates were compared. During the analysis, the A549 cell survival rate was kept constant above 80%. The statistics are shown in Table 2.

### 3.2. Cytotoxicity Analysis

**Figure 4:**
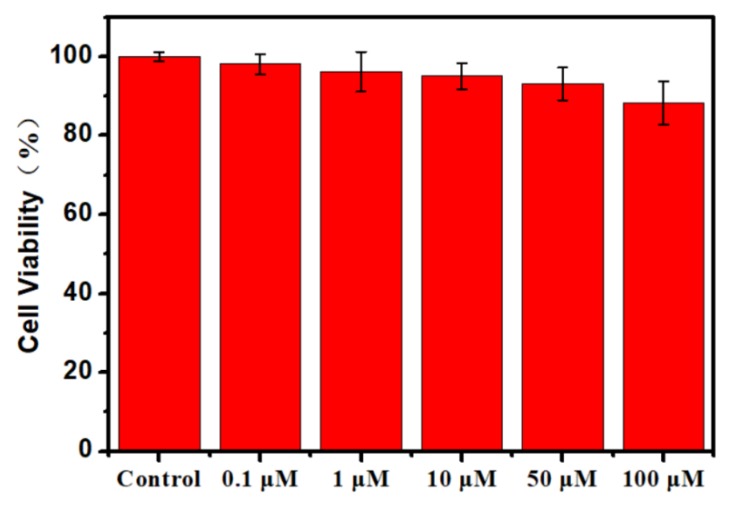
Toxicity test of compound **DY6** and A549 cells after 48 h of co-culture.

The compounds **DY1**–**DY7** were co-cultured with A549 lung cancer cells, and the inhibitors showed low cytotoxicity (Figure 4). The cytotoxicity data of the thiosemicarbazone compounds co-cultured with A549 cells for 48 h and detected by MTT showed that the selected thiosemicarbazone compounds exhibited low toxicity. As the cells were co-cultured for 48 h at a concentration of 100 μM, the cell viability was maintained at 80% or higher, indicating that such inhibitors were less damaging to cells and exhibited low toxicity. To facilitate the comparison of cytotoxicity data, a comparative analysis of the concentrations of different chelators was performed when the A549 cell survival rate was maintained above 80%. The statistics are shown in Table 2.

The effective concentration of **DY1**, **DY2**, **DY4**, and **DY6** was measured up to 100 μM, while **DY3**, **DY5**, and **DY7** showed the same effect on the survival of A549 cells at the same concentration, with an effective concentration of 50 μM. This indicates that, after fiber modification, if the concentration of the thiosemicarbazone compounds was lower than 50 μM, they exhibited low toxicity and it did not affect the normal use of the fibrous material.

### 3.3. EPR Measurements

EPR studies of metal complexes present much useful information about coordination bonding. It is very important to see how the EPR data correlate practically with various structure factors for the geometrical configurations of the coordinating atoms with the copper complexes [23].

**Figure 5:**
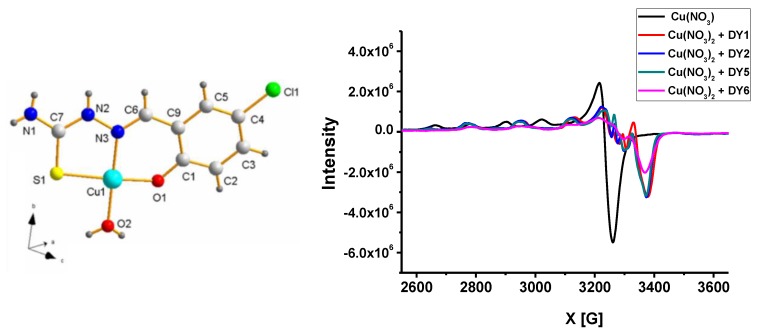
The single-crystal structure of **DY5**–Cu (II) compound (CCDC: 1030350) [15] and the EPR spectrum of the **DY1**–Cu (II), **DY2**–Cu (II), **DY5**–Cu (II), and **6**–Cu (II) complexes (ethanol, 2.5 mM).

The EPR spectrums (Figure 5) suggests that that the curve of Cu(NO_3_)_2_ solution is significantly different from the other four EPR curves, indicating that the addition of the chelating agent changes the chemical environment around Cu(II). The EPR spectra of similar chelating groups and complexes of the same coordination atom with copper ions have a high degree of similarities such as **DY5**–Cu(II), **DY1**–Cu(II), **DY2**–Cu(II), and **DY6**–Cu(II) complex [15]. The EPR curves completely coincided, indicating that the chelating groups of the four ligands are basically the same in the molecular formula compared with the single-crystal structure of the known **DY5**–Cu(II) complex. Based on these **DY**-series compounds and Cu (II), it is inferred that the atoms participating in the coordination during forming a complex are also four atoms of 1N-2O-1S, and their coordination modes are basically the same.

### 3.4. Potentiometric Titration

Tyrosinase is a typical copper-containing oxidoreductase, and the catalytically active center is composed of two copper-containing ion sites. The interruption of the binding of copper ions can lead to the loss of their catalytic activity [24,25,26]. The small molecules of thiourea obtained in this work have significant inhibitory effects on tyrosinase. The inhibition may work mainly by the substitution of tyrosinase to catalyze the hydroxide-bridging ligand between the two copper ions in the active center, thereby forming a strong bond the radical site of the microorganism and inhibiting tyrosinase activity [27]. The binding mode of the thiosemicarbazone compounds to the metal ion can be simulated by potentiometric titration experiments.

The dissociation of protons from the as-synthesized thiosemicarbazones followed by pH-potentiometry. Unlike the simplest α-N-heterocyclic thiosemicarbazones, the presence of amino, sulfhydryls, and methyl substituents at different locations in the structure exerted a distinct influence on the proton dissociation constants (p*K_a_*). The increasing numbers of electron-donating amino groups could significantly increase the p*K_a1_*, as a result of sulfhydryl’s or amine’s deprotonation, and slightly decrease the p*K*_a2_ due to the deprotonation of phenols.

**Figure 6:**
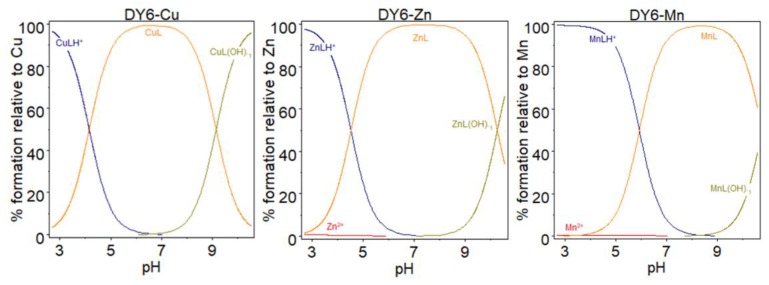
The distribution plots of species measured with the help of potentiometric titrations for the **DY6**–Cu(II), **DY6**–Zn(II) and **DY6**–Mn(II) systems. The **DY6** compound solution was used for potentiometric titrations along with equimolar amounts of Cu(NO_3_)_2_, Zn(NO_3_)_2_, and Mn(NO_3_)_2_ at 25 °C (*I* = 0.1 M KNO_3_).

Figure 6 shows that the complexes mainly exist in the form of CuLH, ZnLH, and MnLH, when **DY6** is mixed with Cu^2+^, Zn^2+^, and Mn^2+^, respectively, at low pH. However, with increasing pH, deprotonation occurred, and the formation of derivatives such as CuL, ZnL, and MnL appeared at a pH 3–10, pH 4–10, and pH 4–10, respectively. Thus, the system mainly existed in the form of CuL, ZnL, and MnL. At higher pH, OH- bound Cu^2+^, Zn^2+^, and Mn^2+^ appeared, which were mainly CuL(OH), ZnL(OH), and MnL(OH).

Both the cumulative stability constant and the stepwise stability constants were calculated. The binding constants between **DY6** and Cu^2+^, Zn^2+^, and Mn^2+^ were 20.15, 14.07, and 14.37, respectively.

The binding constants between thiosemicarbazone compounds **DY1**–**DY7** and metal ions were all greater than 10^12^ M^−1^, as shown in Table 3, indicates that the designed compounds have the strong chelating ability. If the binding constant between the chelating agent and Cu^2+^ is greater than 10^18^ M^−1^, which is five orders of magnitude larger than the binding constant between the tyrosinase protein body and the copper ion of 10^13^ M^−1^ [28], then the chelating agent may have captured the Cu^2+^. The ability to capture Cu^2+^ and stabilized its presence in lysin suggests a possibility for chelating of tyrosinase central metallic ions in cells and lead to inhibit tyrosinase activity.

### 3.5. X-ray Photoelectron Spectroscopy (XPS)

**Figure 7:**
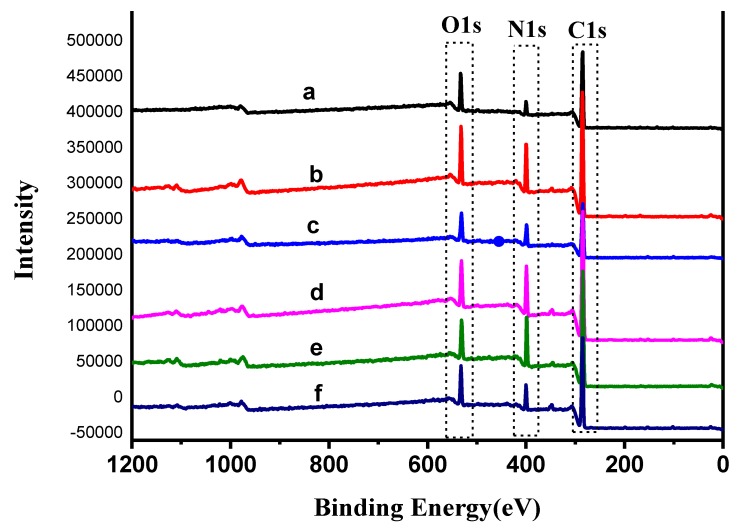
XPS spectra of: (**a**) Original PAN fiber; (**b**) **DY1**–PAN fiber; (**c**) **DY2**–PAN fiber; (**d**) **DY4**–PAN fiber; (**e**) **DY5**–PAN fiber; and (**f**) **DY6**–PAN fiber.

Figure 7 and Table 4 show XPS spectra and atomic concentration of PAN fiber before and after modification. It can be seen that, after protein grafting, it is converted into a carboxyl group (–COOH) by partial hydrolysis of the cyano group on the surface of the acrylic fiber. The abundant amino and amine groups (–NH_2_ and –NH–) on the thiosemicarbazone compounds of the branches lead to a significant increase in the relative content of N (nitrogen) element in the sample after grafting, while C (carbon) and O (oxygen) elements are significantly reduced. This test quantitatively analyzed and proved that the graft modification method of the thiosemicarbazone coupled PAN fiber of the present invention is successful.

### 3.6. Scanning Electron Microscopy (SEM)

**Figure 8:**
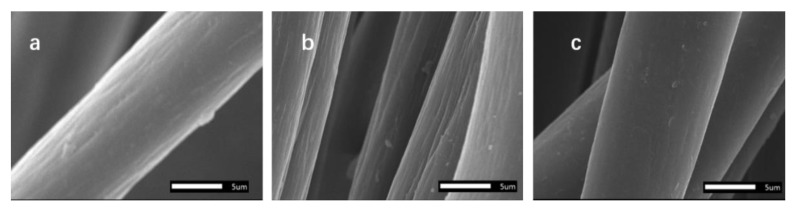
SEM micrographs of the external surface of: (**a**) original PAN fibers; (**b**) hydrolyzed PAN fibers; and (**c**) grafted **DY6**–PAN fibers.

The activity, toxicity, and potentiometric titration tests showed that compound **DY6** exhibited the most optimal performance; therefore, it was chosen for grafting experiments. The SEM micrographs of the original, hydrolyzed, and grafted PAN fibers were recorded, and the results are presented in Figure 8. It can be seen that the surface of the PAN fibers has a certain smooth groove, whereas the surface of the hydrolyzed PAN fibers has obvious gully cracks in the original groove, and the surface groove is widened. This indicates that, when the PAN fibers hydrolyzed with NaOH, the surface of the PAN fibers were etched, which is also consistent with the decrease in the strength of the fibers after hydrolysis.

Compared with the surface morphology of the hydrolyzed PAN fibers, the surface of the fibers after the treatment with the thiosemicarbazone solution became smooth, the original groove marks and etched lines disappeared, and the fine surface of the fibers was filled, which indicated that a thiosemicarbazone film formed on the surface. These changes further support the change in the breaking strength of the acrylic fibers after hydrolysis grafting.

### 3.7. Grafting Efficiency

Enhancing graft yield of polymers and small molecules via graft copolymerization was one of the effective approaches to improve the modification and functionalization for PAN fiber. Therefore, grafting efficiency directly influenced the success of the experiment. Grafting efficiency of PAN fibers before and after **DY6** grafting was determined gravimetrically. The experimental results are shown in Table 5, and analysis demonstrated that the mean graft ratio was 1.92%.

### 3.8. Mechanical Property Analysis of Fibers

**Figure 9:**
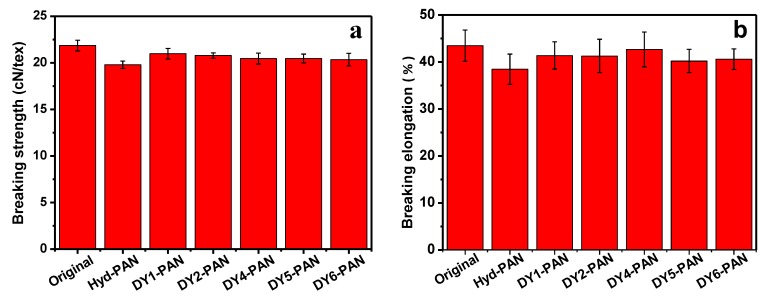
Comparison of breaking strength (**a**) and breaking elongation (**b**) of original PAN fiber, Hyd–PAN fiber, and **DY1**-, **DY2**-, **DY4**-, **DY5**-, and **DY6**-modified PAN fiber.

Both the breaking strength and breaking elongation of the PAN fibers after hydrolysis were decreased, and breaking strength and breaking elongation of the fibers after branching by small molecules increased (Figure 9 and Table 6). Combined with the analysis in Figure 8, the outer surface of the PAN fibers before hydrolysis was smooth, and, after hydrolysis, the fine cracked portion was etched away, the smooth outer surface became rough, and deep cracks and holes appeared. The mechanical properties of the fibers after hydrolysis were reduced compared to the original fibers before hydrolysis, in terms of both breaking strength and elongation at break. This occurred because the PAN fibers were hydrolyzed. On the one hand, a large number of strongly polar –CN groups on the surface of the fibers were converted into less polar –COOH and –CONH_2_ groups, which weakened the PAN macromolecular chain, and the interaction caused the strength to decrease. On the other hand, the hydrolysis also caused the surface of the fibers to be continuously etched, so that the cracks and voids on the surface of the fibers continuously increased, leading to a decrease in fiber strength. However, the breaking strength of the fibers after grafting was significantly higher than that of the hydrolyzed fibers, which was almost similar to the original fibers. This occurred because, after the small molecules were grafted on the surface of the PAN fibers, the surface of the fibers not only became completely covered by a layer of small molecular film, but it also better filled the surface etching, cracks, and voids caused by hydrolysis of the fibers.

The breaking elongation strength at break of the fibers was increased (Table 6). It could be seen that cracks and voids appearing on the surface of the hydrolyzed fibers were almost completely covered in the grafted fibers. This indicates that grafting a small molecule of thiosemicarbazone on the surface of PAN fibers can not only provide a relatively complete small molecular coating on the surface of the fibers, but it also contributes certain reinforcement and modification to the surface defects caused by hydrolysis of the PAN fibers. This function can better compensate for defects such as surface damage and deficient mechanical properties of the fibers due to hydrolysis. As a result, the excellent strength of the original PAN fibers is retained, and the superiority in fiber elasticity and flexibility is demonstrated with the graft of thiosemicarbazone onto the original ordinary PAN fibers.

### 3.9. FTIR Spectroscopy

**Figure 10:**
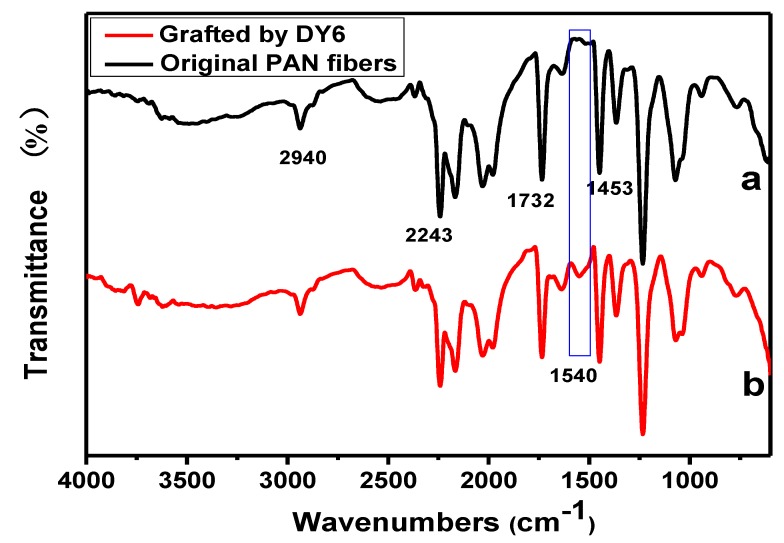
FTIR spectra of: (**a**) original PAN fibers; and (**b**) grafted **DY6**–PAN fibers.

Figure 10 depicts the FTIR absorption spectra of the original PAN fibers (Figure 10a) and the **DY6**-modified PAN fibers (Figure 10b). The FTIR spectra of the original PAN fibers show the characteristic peaks at 2243 (γ C≡N), 1732 (γ C=O), and 1453 (δ CH_2_) cm^−1^, where γ represents a stretching vibration, and δ denotes a bending vibration. The absorption peaks of these characteristics are still present in the thiosemicarbazone graft sample, indicating that the method of small molecule branching treatment of thiosemicarbazone does not destroy or weaken the original molecular structure of the PAN fibers and that some features of the original PAN fibers are preserved. New absorption peaks were displayed at ~1540 (δ N–H and υ C–N, amide II) and ~3500 (δ –OH) cm^−1^, which indicate that the molecular modification of the surface of PAN fibers by thiosemicarbazone is feasible. The IR spectra further prove that modified acrylic fibers were successfully obtained.

### 3.10. Antibacterial Test

**Figure 11:**
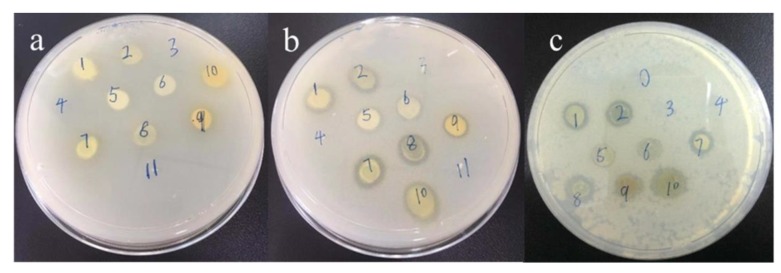
Antibacterial effect of different compounds on different bacteria: (**a**–**c**) the bacteriostatic effects of different compounds on *E. coli*, *S. aureus,* and *B. subtilis.* (Note: Marks 1, 3, 5, 7, and 9 on the plates are **DY1**, **DY2**, **DY4**, **DY5**, and **DY6** with concentrations of 20 g/L; Mark 2, 4, 6, 8, and 10 on the plates are **DY1**, **DY2**, **DY4**, **DY5** and **DY6** with concentrations of 10 g/L; and Marks 0 and 11 on the plates are equal volume of DMSO solution).

The antibacterial tests on the different compounds with different bacteria (Figure 11a) showed that compounds **DY1**, **DY2**, **DY4**, **DY5**, and **DY6** do not have an antibacterial effect on *E. coli.* Figure 11b,c indicates that the zones of inhibition appeared on the samples of Marks 1 and 2 (**DY1**), Marks 5 and 6 (**DY4**), Marks 7 and 8 (**DY5**), and Marks 9 and 10 (**DY6**); the results state that the compounds of **DY1**, **DY4**, **DY5**, and **DY6** express good antibacterial activities on both of *S. aureus* and *B. subtilis*.

**Figure 12:**
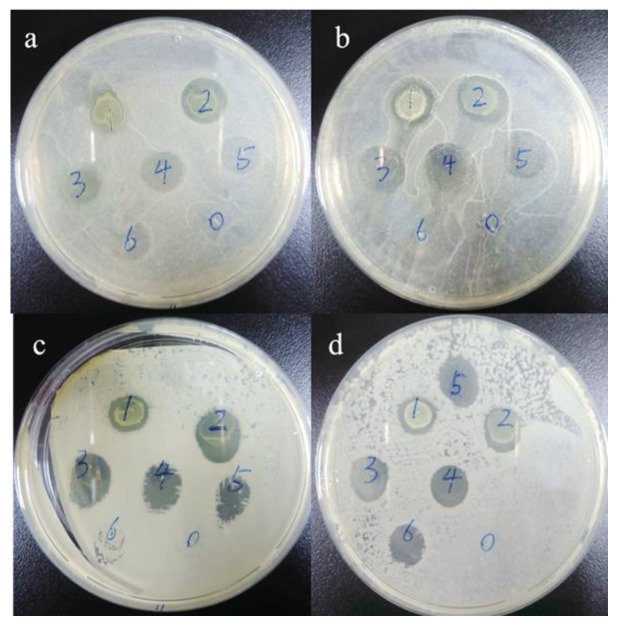
Antibacterial effects of different thiourea molecules: (**a**,**c**) the inhibitory effect of **DY1** at different concentrations on *S. aureus* and *B. subtilis*, respectively; and (**b**,**d**) the inhibitory effects of **DY6** at different concentrations on *S. aureus* and *B. subtilis*, respectively. (Mark 1, 10 g/L; Mark 2, 5 g/L; Mark 3, 2 g/L; Mark 4, 1 g/L; Mark 5, 0.5 g/L; Mark 6, 0.2 g/L; and Mark 0, DMSO).

As shown in Figure 12 and Table 7, the **DY1** and **DY6** showed a strong antibacterial activity against *S. aureus* and *B. subtilis* at a concentration of 0.02 µM. **DY1** exerted bacterial inhibition effect against both microorganisms at a concentration of 0.008 µM. The antibacterial effect is mainly attributed to the presence of phenol groups in the **DY6** structures. The substituents such as methyl and diethylamino groups in **DY1** and **DY6** enhanced the affinity of cell due to the electron effect, which provided better antibacterial effect against both tested microorganisms.

**Figure 13:**
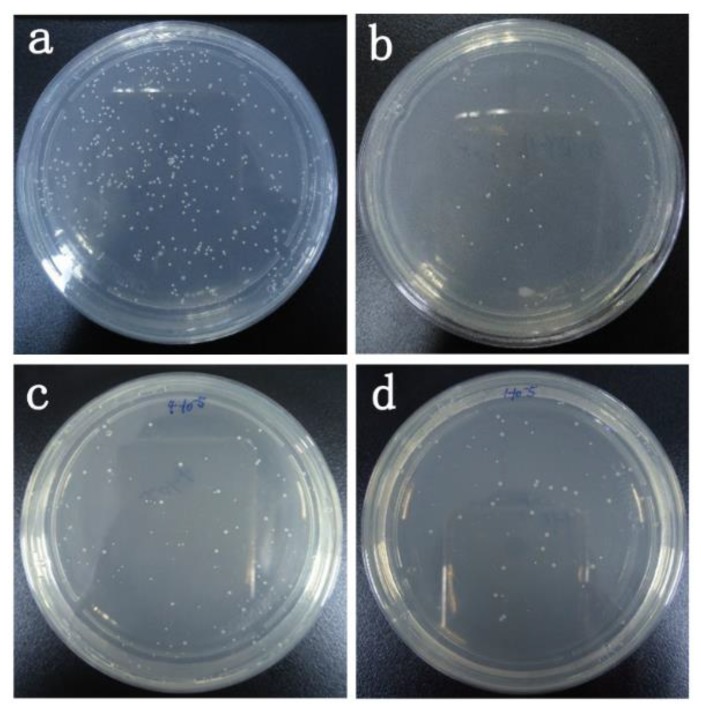
Antibacterial effect against *S. aureus*: (**a**) control sample; (**b**) after treatment with **DY6** ay the concentration of 1.0 µM; (**c**) original PAN fibers; and (**d**) grafted **DY6**–PAN fibers.

As shown in Figure 13, the **DY6** sample and the PAN fibers treated with **DY6** presented a considerably lower number of colony-forming bacterial units at an inhibition effect of 88.6% and 45.1%, respectively. These observations clearly confirmed a very strong inhibitory effect of **DY6**. The bacterial inhibition effect of the synthesized compound mainly originated from the lipophilic characteristics of the thioamide synthon substituted aryl ring, which crossed the cell membrane of the microorganism, and, thus, excellent antibacterial activity was exerted against the tested bacterial strain.

## 4. Conclusions

Seven structurally representative thiourea molecules were synthesized using condensation reaction with thiosemicarbazone and different substituents of benzaldehyde. The bioactive screened thiourea molecule was covalently coupled with hydrolyzed PAN fibers. Characterizations by infrared spectroscopy, scanning electron microscopy and X-ray photoelectron spectroscopy found that the thiourea molecules (**DY6**) were successfully coated on the PAN fibers. The modified PAN fibers not only maintained mechanical strength but also inhibited tyrosinase activity, which could provide anti-browning function for fruits and vegetables packaging economically at the industrial scale.

## Figures and Tables

**Table 1:** ESI-MS spectral data of thiosemicarbazone compounds.

Complex	AtomicMass	Atomic Mass Calculated				Atomic Mass Found				Color	Yield (g)
		C	H	N	S	C	H	N	S		
**DY1** (C9H11N3OS)	210.05	51.66	5.30	20.08	15.32	51.60	5.26	20.11	15.24	White crystal	1.39 (66%)
**DY2** (C8H9N3O2S)	212.05	45.49	4.29	19.89	15.18	45.55	4.24	19.93	15.23	Light yellow	1.14 (59%)
**DY3** (C8H8N4O3S)	241.05	40.00	3.36	23.32	13.35	39.89	3.38	23.40	13.29	Yellow solid	1.41 (63%)
**DY4** (C9H11N3O2S)	226.08	47.99	4.92	16.65	14.23	47.84	4.98	18.54	14.20	White solid	1.35 (61%)
**DY5** (C8H8ClN3OS)	230.00	41.84	3.51	18.30	13.96	41.75	3.59	18.25	14.03	White crystals	1.60 (71%)
**DY6** (C12H18N4OS)	267.15	54.11	6.81	21.03	12.04	54.14	6.79	21.10	12.09	Light brown	1.78 (69%)
**DY7** (C9H11N3OS)	238.06	51.66	5.30	20.08	15.32	51.62	5.25	20.12	15.29	White solid	1.32 (69%)

**Table 2:** Statistical analysis of IC_50_ values for semi-inhibitory concentrations of tyrosinase and comparison of toxicity data for thiosemicarbazone on A549 cells.

Compounds	IC_50_ (μM)	Concentration of Compounds When 80% of Cells Survive (μM)
**DY1**	1.88	<100 μM
**DY2**	4.75	<100 μM
**DY3**	9.88	<50 μM
**DY4**	1.12	<100 μM
**DY5**	1.68	<50 μM
**DY6**	0.60	<100 μM
**DY7**	41.2	<50 μM

**Table 3:** The statistical comparison of coordination with respect to the selected divalent metal ions.

Ligand	DY1	DY2	DY3	DY4	DY5	DY6
log*K*_Cu_^2+^, n = 1, M^−1^	19.15 ± 0.11	18.83 ± 0.14	19.10 ± 0.15	18.95 ± 0.10	19.43 ± 0.22	20.15 ± 0.14
log*K*_Zn_^2+^, n = 1, M^−1^	13.13 ± 0.21	13.53 ± 0.11	13.69 ± 0.12	13.56 ± 0.17	12.51 ± 0.20	14.07 ± 0.37
log*K*_Mn_^2+^, n = 1, M^−1^	13.67 ± 0.31	14.27 ± 0.17	14.48 ± 0.11	14.01 ± 0.08	13.65 ± 0.16	14.37 ± 0.30

**Table 4:** Surface element content and binding energy of PAN fiber before and after modification.

Fibers	Atomic Concentration (%)			Binding Energy/eV		
	C1_S_	N1_S_	O1_S_	C1_S_	N1_S_	O1_S_
Original PAN fiber	78.75	7.22	13.50	284.80	399.35	532.37
**DY1**–PAN fiber	73.45	14.49	11.68	284.81	399.01	530.96
**DY2**–PAN fiber	73.22	13.78	12.74	284.80	398.20	530.86
**DY4**–PAN fiber	75.86	14.09	9.54	284.81	398.93	531.04
**DY5**–PAN fiber	74.14	16.8	8.79	284.80	398.29	530.43
**DY6**–PAN fiber	77.70	10.96	10.96	284.80	399.44	531.64

**Table 5:** Grafting efficiency of PAN fibers before and after **DY6** grafting.

Grafted (g)	Original (g)	Grafting Efficiency (%)
0.4888	0.4796	1.90
0.4884	0.4798	1.89
0.4889	0.4791	2.00
0.4892	0.4794	2.00
0.4881	0.4793	1.80
**Mean**		1.92 ± 0.08

**Table 6:** Comparison of breaking strength and elongation of PAN fiber before and after modification.

Sample.	Breaking Strength (cN/Tex)	Breaking Elongation (%)
Original PAN fiber	21.85	43.48
Hydrolyzed PAN fiber	19.79	38.47
**DY1**	20.98	41.38
**DY2**	20.78	41.29
**DY4**	20.45	42.68
**DY5**	20.46	40.21
**DY6**	20.34	40.60

**Table 7:** Comparison of bacteriostatic effects of different thiourea molecules.

Bacteria	Thiourea Molecules	Concentration (μM)
*S. aureus*	**DY1**	0.02
	**DY6**	0.02
*B. subtilis*	**DY1**	0.008
	**DY6**	0.008
